# Supplementary material for: Processed and ultra-processed foods are associated with high prevalence of inadequate selenium intake and low prevalence of vitamin B1 and zinc inadequacy in adolescents from public schools in an urban area of northeastern Brazil
Source: PLoS One. 2019 Dec 4;14(12):e0224984. doi: 10.1371/journal.pone.0224984 (PMC6892533; doi:10.1371/journal.pone.0224984)
Supplement: S1 Table — aAdapted from Louzada et al.[37]. (DOCX) [file pone.0224984.s001.docx]

**S1 Table**

Classification of foods and ingredients according to their industrial processing characteristics

| Food groups and consumable items^a^ | Description |
| --- | --- |
| *Processed* |  |
| French Bread | Bread made with wheat flour, water, yeast, and salt (but not containing other substances such as hydrogenated fat and chemical additives) |
| Cheeses | Cheeses made of milk and salt |
| Processed meats | Salty meats, canned fish, and fish preserved in oil |
| Canned fruits and vegetables | Canned vegetables, maize or peas, fruit in syrup, and candied fruit. |
| *Ultra-processed* |  |
| Cakes, pies, and cookies | Products baked with ingredients that include substances such as hydrogenated vegetable fat, sugar, starch, whey, emulsifiers, and other additives |
| Fast food dishes | Hamburgers of all kinds, hot dogs, and fried and baked snacks, or other similar foods. |
| Sugar-sweetened beverages | Soft drinks, artificial juice powder, and carton-packed juices |
| Sliced breads | Sliced breads, processed hamburger or hot dog breads, sweet rolls, and other breads with chemical additives |
| Bakery products | Processed sweets and sweets in general (sweets, ice cream, chocolates, gelatin, and pudding) |
| Snacks | Snack food and maize snack in the form of chips |
| Ultra-processed meats | All kinds of sausages and frankfurters (made of chicken, pork and mixed), mortadella, and ultra-processed meats |
| Meals ready or semi-ready for consumption | Pizzas, frozen pasta or meat dishes, instant noodles, and instant soups |
| Sweetened milk drinks | Sweetened and flavored milk drinks |
| Other ultra-processed foods | Margarine, processed sauces, cereal bars, and breakfast cereals |

^a^Adapted from Louzada et al. ^[37]^.
