# Supplementary material for: Processed and ultra-processed foods are associated with high prevalence of inadequate selenium intake and low prevalence of vitamin B1 and zinc inadequacy in adolescents from public schools in an urban area of northeastern Brazil
Source: PLoS One. 2019 Dec 4;14(12):e0224984. doi: 10.1371/journal.pone.0224984 (PMC6892533; doi:10.1371/journal.pone.0224984)
Supplement: Attachment — Submitted filename: PLOS ONE comments_PONE-D-19-18574.docx [file pone.0224984.s002.docx]

**Processed and ultra-processed foods are associated with high prevalence of inadequate**

**selenium intake and low prevalence of vitamin B1 and zinc inadequacy in adolescents from**

**public schools.**

The study addresses an interesting and relevant subject showing the association of consumption of processed and ultra-processed foods with micronutrient inadequacy in adolescents. However, the MS lacks logical presentation of information hence is difficult to understand.

The introduction doesn’t contain the relevant background information to guide readers to the rest of the manuscript.

The justification or motivation to do this research is not well formulated.

The language not clear.

The outcome of this study mainly depends on the quality of the food composition table used to convert into nutrient intake of the present study subjects. However, this may not work for some nutrients such as selenium. This is because; selenium concentration in foods is mainly determined by the amount of the element in the soil which is quite variable in short distances. Thus, data from food composition is not reliable.

Parts of the methodology lack clarity and are not detailed.

The authors in general tried to show the effect of level of processing on micronutrient intake inadequacy that consumption of processed foods was associated with lower intake of selenium but not Zn and B1. Processed foods in general are poor in micronutrient content. However, it is failed to identify whether foods in the present study were enriched with Zn and vitamin B1 concentration.

For example,

*Line 48-50*, Energy consumption from processed foods was associated with higher prevalence of inadequate selenium intake (p < 0.01) but lower prevalence of inadequate vitamin B1 intake (p = 0.04).

*Line 50-54*, Energy consumption from ultra-processed foods was associated with lower prevalence of inadequate zinc and vitamin B1 intake (p < 0.01 and p =0.03, respectively). An increase in the proportion of energy obtained from processed and ultra-processed foods may reflect higher prevalence of inadequate selenium intake and lower prevalence of vitamin B1 and zinc inadequacy.

*Line 74-75*. It is not always true to claim that sedentary behaviors among adolescents lead to greater consumption of packed or ready to eat foods.

**Methodology**

*Line 136-140*: Ethical consideration/approval has to be presented separately

*Line 141-148*: The inclusion and Inclusion criteria may work for the main study “Risk factors for cardiovascular disease among adolescent beneficiaries of the Brazilian government National School Lunch Program (PNAE)” but not for this specific MS.

The sample size was determined considering altered lipid profile as an outcome which is not consistent with the present study outcome.

Weight and height measurement procedure is not detailed

*Line 179:* ‘The morning was chosen to more easily enable methodological procedures’. This is not clear. Does this mean ‘anthropometric measurement was done during the morning? Why?

Line 182-185: It is not clear the importance of assessing pubertal stage given the age of study participants was known for this particular study.
